# *CmWOX2* modulates somatic embryogenesis in Chinese chestnut (*Castanea mollissima* Blume)

**DOI:** 10.5511/plantbiotechnology.24.0527a

**Published:** 2024-12-25

**Authors:** Zhilin Sun, Bing Liu, Yuzhen Tian, Xiaowei Li, Yuyuan Long, Qingrong Zhang, TingTing Xiao, Qingqin Cao

**Affiliations:** 1 College of Biological Sciences and Technology, Beijing Forestry University, Beijing 100083, China; 2 College of Plant Science and Technology, Beijing Key Laboratory for Agricultural Application and New Technique, Beijing University of Agriculture, Beijing 102206, China

**Keywords:** Chinese chestnut, *CmWOX2*, functional research, somatic embryogenesis

## Abstract

Chinese chestnut (*Castanea mollissima* Blume) is distinguished by its remarkable nut quality and robustness against disease and environmental stressor. However, its somatic embryogenesis process is notably slow, presenting a significant bottleneck in its cultivation. This study focuses on the *WUSCHEL* (*WUS*)*-related homeobox 2 gene* (*WOX2*), a member of *WOX* transcription factors gene family, known for its critical role in the somatic embryo development of Arabidopsis. We have identified and explored the function of a *WOX2* homolog in Chinese chestnut, termed *CmWOX2*, in the context of somatic embryogenesis. Our analysis revealed seven *WUS* gene family members in the species, with *CmWOX2* being uniquely upregulated in callus. Our experiments demonstrated that suppression of *CmWOX2* expression diminishes somatic embryo production, whereas its overexpression enlarges the embryonic callus diameter. Notably, *CmWOX2* expression levels are threefold higher in varieties with high embryogenic competence, such as ‘Jingshuhong’ and ‘Huaihuang’, compared to those with lower competence, including ‘Jiujiazhong’ and ‘Shandonghongli’. These findings underscored the pivotal role of *CmWOX2* in the initial stages of Chinese chestnut somatic embryogenesis, highlighting its potential as a target for enhancing somatic embryogenesis in this species.

## Introduction

The Chinese chestnut (*Castanea mollissima* Blume) is a versatile tree, holding both economic significance and ecological importance. In the global chestnut production landscape, Chinese chestnut stands out as the highest yield variety (FAOSTAT Statistics Database, http://www.fao.org/faostat/en/#home (Accessed Jul 11, 2023)). Its remarkable adaptability to poor soil and robust resistance to diseases, such as chestnut blight, a devastating fungal disease primarily affecting chestnut trees (Xing et al. 2019), make it a crucial player within *Castanea spp*., contributing to the improvement of exceptional germplasm (Miller et al. 2014). However, the advances of germplasm improvement in Chinese chestnuts has been limited due to the prolonged time consumption for the breeding process.

Somatic embryogenesis (SE) serves as a solution to the constraints of traditional breeding, facilitating the efficient propagation of clonal seedlings and improving germplasm (Wang et al. 2021). Furthermore, SE provides an excellent platform for genetic transformation, mitigating the risk of developing chimeras (Raza et al. 2019; Vidal et al. 2010). Notably, SE-based regeneration systems have been successfully established within *Castanea* genus, encompassing American chestnut (*C. dentata*) (Andrade and Merkle 2005; Xing et al. 1999), European chestnut (*C. sativa*) (Corredoira et al. 2003) and Chinese chestnut (Li et al. 2022; Lu et al. 2017). However, the somatic embryo development process in Chinese chestnut is relatively slow, and the delayed growth of somatic embryos poses a significant bottleneck for the application of genetic transformation and clonal propagation in Chinese chestnut. Therefore, improving the embryogenic competence and increasing the reproduction efficiency of somatic embryos in Chinese chestnut is crucial for its breeding process.

The *WOX* gene family plays a crucial role in various developmental processes, including stem cell repair, organ formation, seed formation, organ regeneration and SE (Hao et al. 2019). The *WUSCHEL (WUS)-related homeobox 2* gene (*WOX2*) is particularly active during early somatic embryo development (Haecker et al. 2004). Despite its potential to enhance embryogenic competence, WOX2 has not yet been identified in chestnut. Hence, we set out to identify the chestnut *WOX2* gene and evaluate its impact on chestnut SE. Phylogenetic reconstruction of the Arabidopsis and chestnut *WOX* genes, along with expression analysis of the chestnut genes in embryogenic callus and developing somatic embryos (Li et al. 2022), indicated that Cm07G01544 is the homology of *AtWOX2*, and we named it *CmWOX2*. Consequently, we investigated the function of *CmWOX2* in regulating SE process via knockdown its expression and overexpression during early somatic embryo development. Results confirmed that *CmWOX2* influences the embryogenic competence of callus by impacting the somatic embryo size. Thus, *CmWOX2* emerges as a potential candidate for improving the embryogenic competence and increasing the reproduction efficiency of somatic embryos in Chinese chestnut.

## Materials and methods

### Plant materials

Immature embryos of *C. mollissima* cv. ‘Yanshanhongli’, ‘Jiujiazhong’, ‘Jingshuhong’, ‘Huaihuang’, and ‘Shandonghongli’ were utilized as a source of explants. The embryogenic callus of *C. mollissima* cv. ‘Yanshanhongli’, utilized for genetic transformation, originated from immature embryos (Lu et al. 2017). *C. mollissima* cv. ‘Jiujiazhong’, ‘Jingshuhong’, ‘Huaihuang’, and ‘Shandonghongli’ were utilized as select optimum *C. mollissima* varieties lines with high embryogenic competence. These embryogenic calli were cultivated at three-weeks intervals on Embryo initiation medium (E1). E1 consisted of 2.3 g l^−1^ McCown’s Woody Plant Medium salts (WPM) (Phyto Tech, USA), 109 mg l^−1^ Nitsch and Nitsch vitamins (Phyto Tech, USA), 1 g l^−1^ casein (Phyto Tech, USA), 3% sucrose (w/v), 0.3% phytagel (w/v) (Phyto Tech, USA), 1.8 µM 2,4-dichlorophenoxyacetic acid (2,4-D) (Sigma, USA), and 1.1 µM 6-benzylaminopurine (6-BA) (Sigma, USA). Following adjustment of the medium’s pH to 5.5 (Merkle et al. 1991), autoclaving was carried out at 121°C for 21 min.

### Identification of *CmWOXs* in Chinese chestnut genome

The complete genome sequence and annotation files for the Chinese chestnut were obtained by downloading them from the webpage (http://castaneadb.net/). The hidden Markov model (HMM) of the homeodomain (HD) (PF00046) was acquired from the Pfam database. Subsequently, the *CmWOX* family genes were identified within the Chinese chestnut protein database using HMM files through hmm search. Compute pI/MW tool in ExPASy database was used to calculate the biochemical parameters of *CmWOXs*. The *CmWOXs* protein of subcellular localization were predicted through WoLF PSORT tools (https://wolfpsort.hgc.jp/).

### Phylogenetic analysis

The protein sequences of WOX from Chinese chestnut and Arabidopsis were used to create the phylogenetic tree by MEGA-X, employing the neighbor-joining (NJ) method with 1000 bootstrap replications.

### Analysis of gene structure and conserved protein motifs

The exon/intron organization of the *CmWOX* genes in Chinese chestnut was assessed using TBtools. Conserved motifs within *CmWOXs* were identified using MEME (http://meme-suite.org/tools/meme).

### Chromosomal distribution of *CmWOXs*

TBtools was utilized for visualizing the distribution of *CmWOX* genes across Chinese chestnut chromosomes. Gene locations and gene density for each chromosome were obtained from the GFF file, accessible at (http://castaneadb.net/ (Accessed Feb 26, 2023)).

### Transformation of embryogenic callus

The *CmWOX2* RNAi construct was generated by integrating a 300 bp the coding sequence (CDS) of *CmWOX2* driven by the 35S promoter, into the pK7GWIWG2 (II) RR-277 vector, which harbors the *35S*::*DsRED* expression cassette (Figure S2). For the transformation process, CDS of the *CmWOX2* (777 bp) was cloned and inserted into the XbaI/KpnI site of the Super1300 vector. The expression of *CmWOX2* was driven by the 35S promoter, and the *eGFP* reporter gene was fused. The empty Super1300 vector containing the *eGFP* gene served as the control (Figure S1). These constructs were introduced into preserved callus using *Agrobacterium tumefaciens* strain GV3101, following a previously described method (Sun et al. 2020). Approximately 0.5 g embryogenic callus and 5 ml *Agrobacterium* were incubated for 1 h at 21–23°C on a rotating shaker. The calli were then transferred on E1 medium in the dark for 2 days. Subsequently, calli were cultured on E1 medium supplemented with 100 mg l^−1^ cefotaxime and 500 µM timentin in dark for one week. The antibiotic-resistant calli were screened using E1 medium with 100 mg l^−1^ cefotaxime, 500 µM timentin and 80 mg l^−1^ hygromycin (Gao et al. 2020).

### Subcellular localization assay

The constructs (*35S::CmWOX2-eGFP* and *35S::eGFP*) were expressed in tobacco leaf epidermal cells using the transient transformation method (Sparkes et al. 2006). Subsequently, the samples were stained with DAPI to visualizing the nucleus. Following staining, the fluorescence activity of the CmWOX2 protein fused with eGFP and nucleus of the samples were captured using a confocal laser scanning microscope (Leica Stellaris 5). GFP was excited with 488 nm laser and detected at 505 nm–550 nm, while DAPI was excited with 405 nm laser and detected at 450 nm–470 nm.

### Induction and observation of somatic embryos

Embryogenic calli from transgenic lines were separately incubated in E1 liquid medium on a rotary shaker (100 rpm) in darkness at 23–25°C for one week. Then suspension cultures were successively passed through 425 µm and 150 µm mesh size sterile steel sieve. Approximately 0.1 g embryogenic callus on the 150 µm mesh size sieve was transferred to E2 liquid suspension culture and incubated on a rotary shaker (100 rpm) in darkness at 23–25°C for one week. Suspension cultures were passed through 150 µm mesh size sterile steel sieve, removing excess liquid medium, and the total weight was recorded. 0.1 g embryogenic cultures were observed and photographed by a fluorescence Stereomicroscopy (ZEISS SteREO Discovery. V20, Germany) with a GFP filter. The rest of the embryogenic cultures on 150 µm mesh size sieve was transferred to E2 solid medium and incubated in darkness at 23–25°C for two weeks. Stereomicroscopy (ZEISS SteREO Discovery. V20, Germany) was used to observe and photograph somatic embryos, and their diameter was measured using Image J.

The number of embryos from 0.1 g EC was calculated as the total weight of embryogenic cultures divided by 0.1 g, and then multiplied by the number of embryos in 0.1 g embryogenic cultures.

### RNA isolation, cDNA synthesis, and qPCR assays

Approximately 0.1 g transgenic calli were incubated in E1 liquid medium for one week, these transgenic calli were successively passed through the 150 µm mesh size sieve. Total RNA of these transgenic calli were extracted using the Plant RNA Kit (Omega, USA) following the manufacturer’s recommendations, and then RNA samples were digested with DNase I (Takara, Japan) to remove any remaining DNA. cDNA was synthesized using M-MLV reverse transcriptase (Invitrogen, USA). qPCR experiments were conducted using the SYBR Premix Ex Taq II Kit (TaKaRa, Japan) with a CFX96 Touch Real-Time PCR Detection System (Bio-Rad, USA) following the manual’s recommendations. Each reaction was prepared in a total volume of 15.0 µl including 1.0 µl cDNA (∼20 ng), 0.3 µl forward primer (10 µM), 0.3 µl reverse primer (10 µM), 7.5 µl SYBR Premix Ex Taq II, and 5.9 µl double-distilled water. Each reaction was performed in triplicate. The PCR program was conducted with an initial step at 95°C for 3 min, 40 cycles of 95°C for 5 s and 60°C for 30 s. Melting curves were completed by increasing the temperature from 65 to 95°C. The relative expression of SE-related genes was calculated using the 2^−ΔΔCt^ method, with *CmActin* (GenBank accession no. MK038768) as the reference gene (Gao et al. 2020). All primer sequences shown in Table S1.

## Results

### Identification of *CmWOXs* in Chinese chestnut genome and phylogenetic analysis of *CmWOXs*

**Figure 1:**
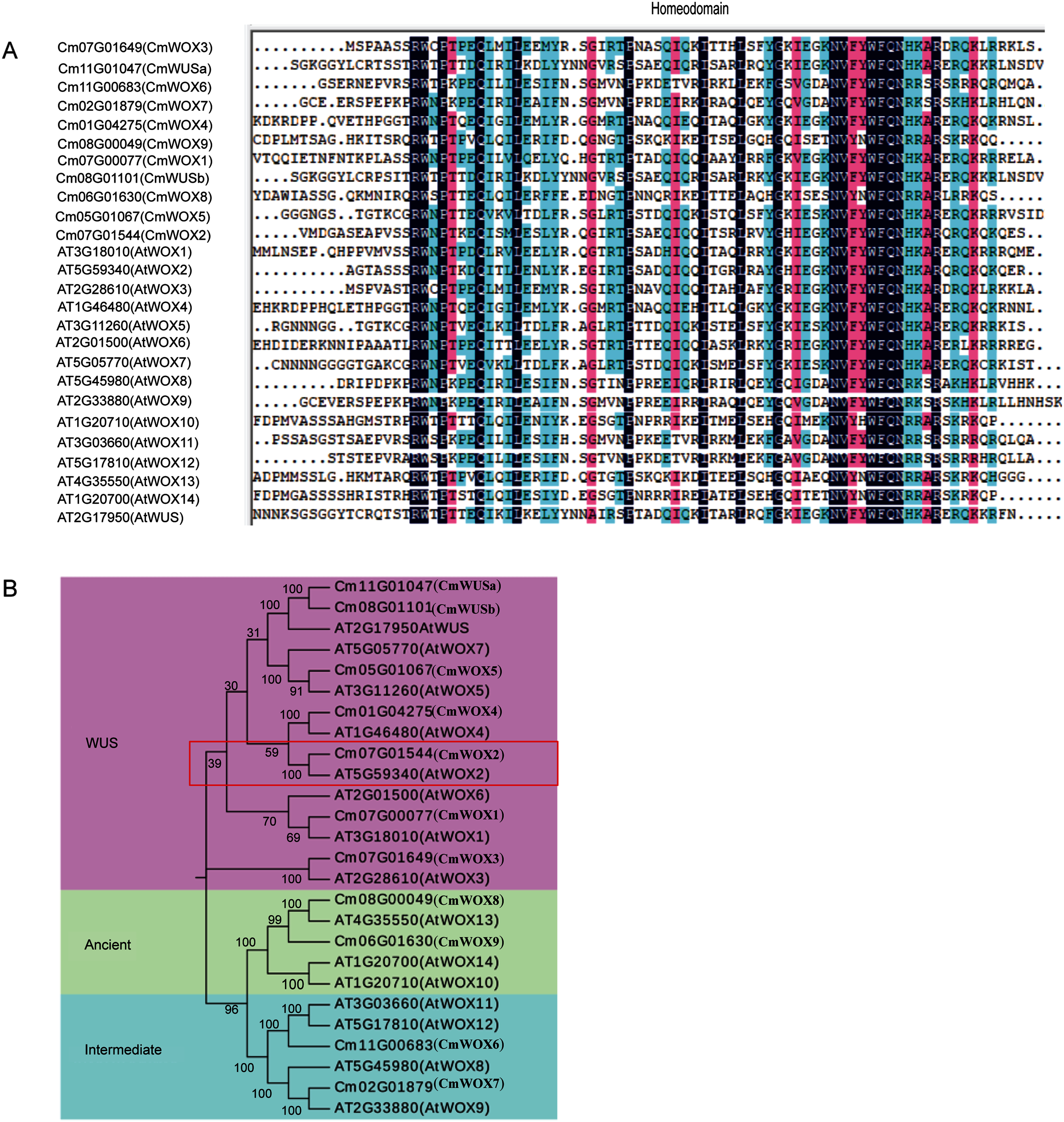
Figure 1. CmWOX protein sequence analysis and phylogenetic tree of the WUS gene family. A. Analysis of CmWOX protein sequence. B. Phylogenetic tree of WUS gene family.

To identify WOX family genes in the Chinese chestnut genome, we employed the Hidden Markov Model (HMM) profile of the homeodomain (HD) (PF00046), as queries for searching against the chestnut database (genome version of *C. mollissima* cv. HBY-2 http://castaneadb.net/ (Accessed Feb 26, 2023)). Additionally, we obtained WOX protein family sequences of *Arabidopsis* (van der Graaff et al. 2009) from the TAIR database. As a result, we identified a total of 11 *WOX* genes in the Chinese chestnut genome (Figure 1A).

Phylogenetic analysis was conducted using protein sequences of CmWOXs through MEGA X. We organized CmWOX sequences alongside well-studied AtWOX protein family members, categorizing them into the corresponding ancient, intermediate and WUS clades. Among these, seven CmWOX members fall into to the WUS clades, two into intermediate clades, and two into ancient clade (Figure 1B). Subsequently, we named the chestnut *WOXs* based on their closely related Arabidopsis homologs (Figure 1B). The phylogenetic analysis suggested that Cm07G01544 (*CmWOX2*) is the closest *AtWOX2* homolog gene and is allocated in the WUS clade.

### Gene structure, conserved motifs analysis and genome distribution of the *CmWOX* gene family members

**Figure 2:**
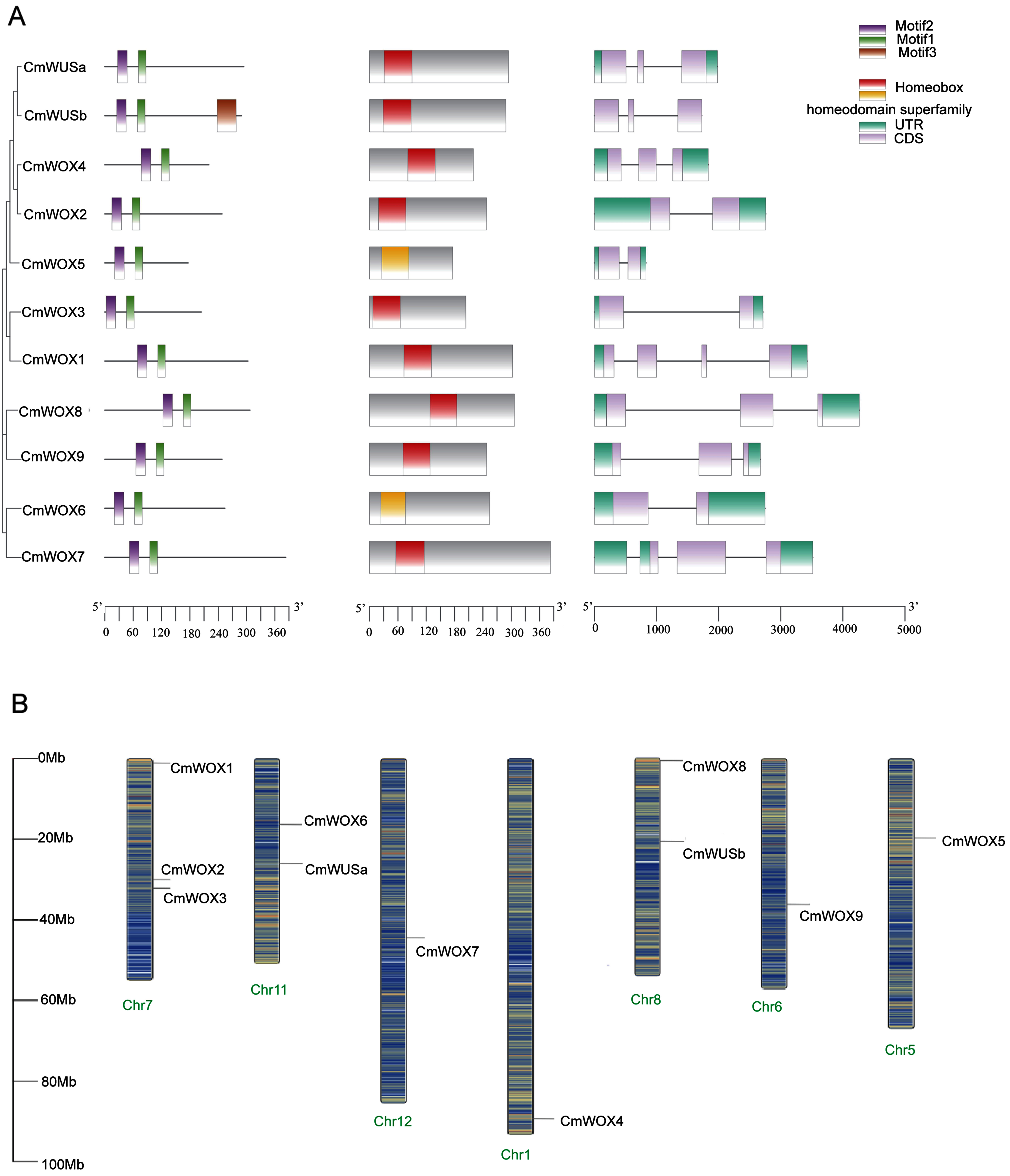
Figure 2. Predicted structures of Chinese chestnut WOX family genes and their genome distribution. A. Exon-intron organization and predicted motifs of Chinese chestnut WOX family genes. B. Distribution of the WUS family gene members in the chromosome of Chinese chestnut genome.

To elucidate the evolution of the *CmWOX* gene family, we conducted analyses of gene structure, conserved motifs, and genome distribution. The lengths of CmWOX sequences varied from 175 (CmWOX5) to 382 (CmWOX7) amino acids. All *CmWOX* genes in Chinese chestnut feature exons and introns, with a maximum of four exons identified (Figure 2A). The intron–exon arrangement of *CmWOX* genes is depicted in Figure 2A. Utilizing the MEME tool, we identified two conserved motifs (Motifs 1 and 2) in each CmWOX sequence, encoding two highly conserved homeodomains (Figure 2A), change to. Notably, CmWUSb contains three motifs (Motifs 1, 2 and 3). It has also been predicted that all the CmWOX proteins are localized in the nucleus (Table 1).

**Table 1:** Table 1. Basic physico-chemical properties of *CmWOX* genes.

Gene ID	CDS Length (bp)	Protein Length (aa)	pI	MW (kDa)	Prediction of subcellular localization
CmWOX1	909	302	9.5	35.21	Nucleus
CmWOX2	744	247	6.71	27.74	Nucleus
CmWOX3	612	203	9	23.87	Nucleus
CmWOX4	660	219	8.63	25.27	Nucleus
CmWOX5	528	175	8.5	20.44	Nucleus
CmWOX6	762	253	5.44	27.78	Nucleus
CmWOX7	1149	382	6.7	42.2	Nucleus
CmWOX8	921	306	5.89	35.13	Nucleus
CmWOX9	744	247	6.67	28.42	Nucleus
CmWUSa	882	293	5.44	32.72	Nucleus
CmWUSb	867	288	6.26	32	Nucleus

The 11 *CmWOX* genes were mapped to 7 chromosomes in the Chinese chestnut Genome (Figure 2B), specifically chromosome 1, 2, 5, 6, 7, 8, and 11. Overall, *CmWOX* genes were evenly distributed across those chromosomes, with the exception of chromosome 7, 11 and 8, which harbored 3, 2 and 2 genes, respectively (Figure 2B).

### Expression profiles of *CmWOX2* during SE in Chinese chestnut

**Figure 3:**
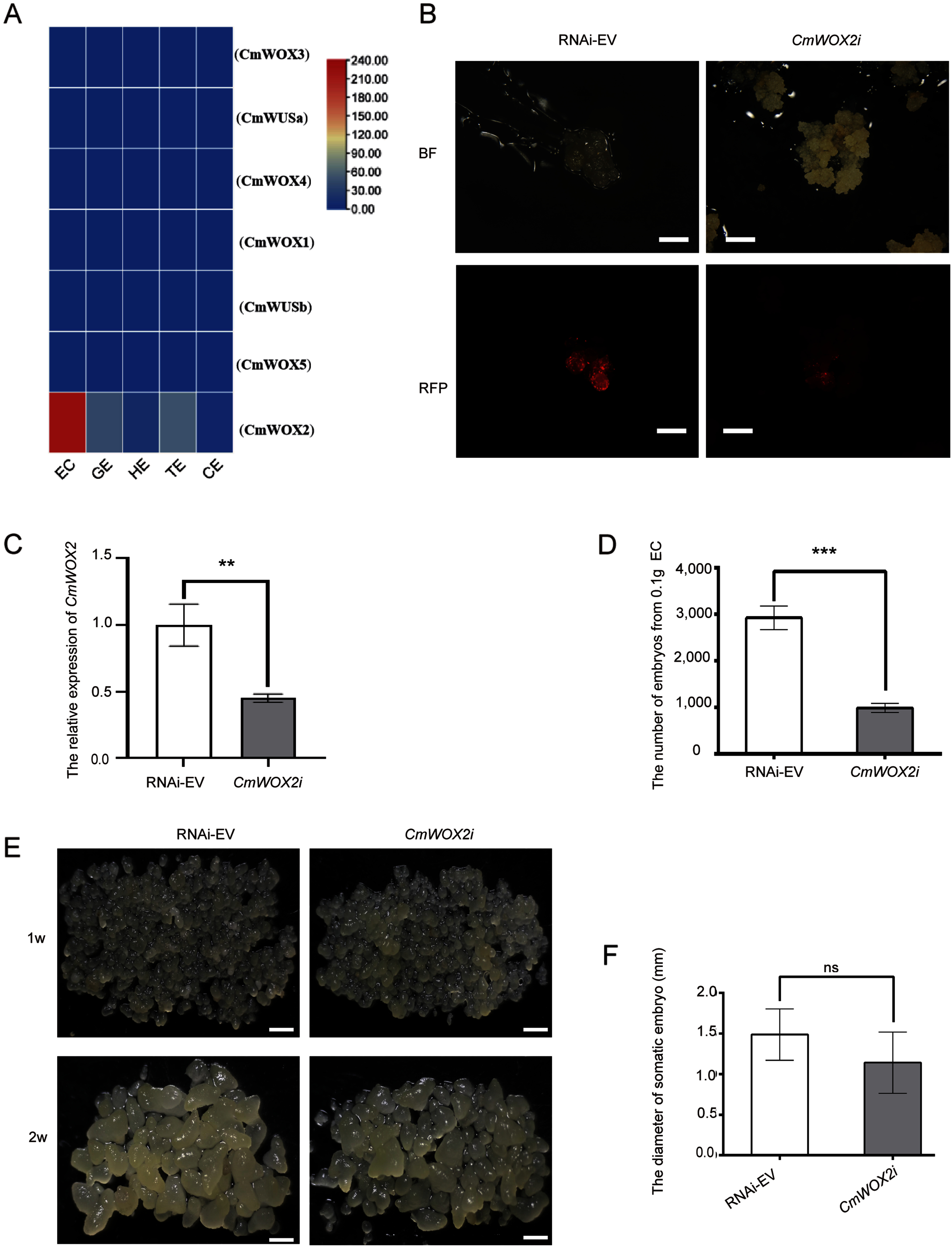
Figure 3. Expression levels of *CmWUS* gene family in different developmental stages of somatic embryos and knockdown of *CmWOX2* expression in Chinese chestnut callus. A. Expression levels of *CmWUS* gene family in different developmental stages of somatic embryos. B. Visualization of *35S::CmWOX2i* and RNAi empty vector control callus expressing *DsRED*. C. Analysis of *CmWOX2* gene expression in *CmWOX2i* and EV callus using qRT-PCR. D. Number of embryos formed from 0.1 g of *CmWOX2i* and EV callus. E. Growth status of *CmWOX2i* and EV callus after two weeks cultivated in E2. F. Diameter of *CmWOX2i* and EV callus after two weeks cultivated in E2. Abbreviations: embryogenesis callus (EC), globular (GE), heart (HE), torpedo (TE), cotyledon-type embryos (CE) and *CmWOX2i* (*CmWOX2*-RNAi). Bars: 500 µm. Data represent mean±S.D. of 3 independent experiments. Student’s *t* test, *** *p*<0.001, ** *p*<0.01, ns indicate no statistically significant differences (*p*<0.05).

In a prior study, we defined the developmental stages spanning from embryogenic callus to somatic embryos, encompassing embryogenesis callus, globular, heart, torpedo, and cotyledon-type embryos, based on morphological difference (Lu et al. 2017). To validate the involvement of Cm07G01544 (*CmWOX2*) in chestnut SE development, we analyzed the gene expression profile of *WUS* clade members using transcriptome data obtained from different somatic embryo stages in Chinese chestnut (Li et al. 2022). The results reveal that *CmWOX2* is the sole gene detected during SE within the *WUS* clade, with high expression levels observed during the embryonic callus stage (Figure 3A). This finding further supports the notion that Cm07G01544 is the closest *AtWOX2* homolog gene and may play a significant role in SE.

### Knockdown of *CmWOX2* reduces the embryogenic capability of Chinese chestnut callus

To further investigate the effect of *CmWOX2* on SE, a *CmWOX2* RNAi construct was generated by inserting a 300 bp CDS of *CmWOX2* driven by the 35S promoter cloned into pK7GWIWG2 (II) RR-277 vector containing *35S::DsRED* as a selection marker, which was then transferred into Chinese chestnut callus. Subsequently, *CmWOX2*-RNAi (*CmWOX2i*) callus lines and RNAi empty vector (EV) lines as controls were generated (Figure 3B).

To validate the RNA interference effects of this construct, *CmWOX2i* and EV callus were analyzed for *CmWOX2* gene expression via qRT-PCR. The results showed that the expression level of *CmWOX2* in the *CmWOX2i* callus was 50% lower than in the EV calli (Figure 3C). Then, we analyzed the development of the *CmWOX2i* and EV embryogenic callus cultivated with E2. We found that the number of embryos from 0.1 g of embryogenic callus was significantly lower in *CmWOX2i* lines compared with the EV lines after two weeks. Additionally, the average diameter of *CmWOX2i* somatic embryo was smaller than EV control, although the difference did not reach statistical significance with *p*<0.05 (Figure 3D, E). These findings suggests that *CmWOX2* knockdown reduces the embryogenic capability of Chinese chestnut callus.

### Overexpression of *CmWOX2* enhances the embryogenic competence of the Chinese chestnut callus

**Figure 4:**
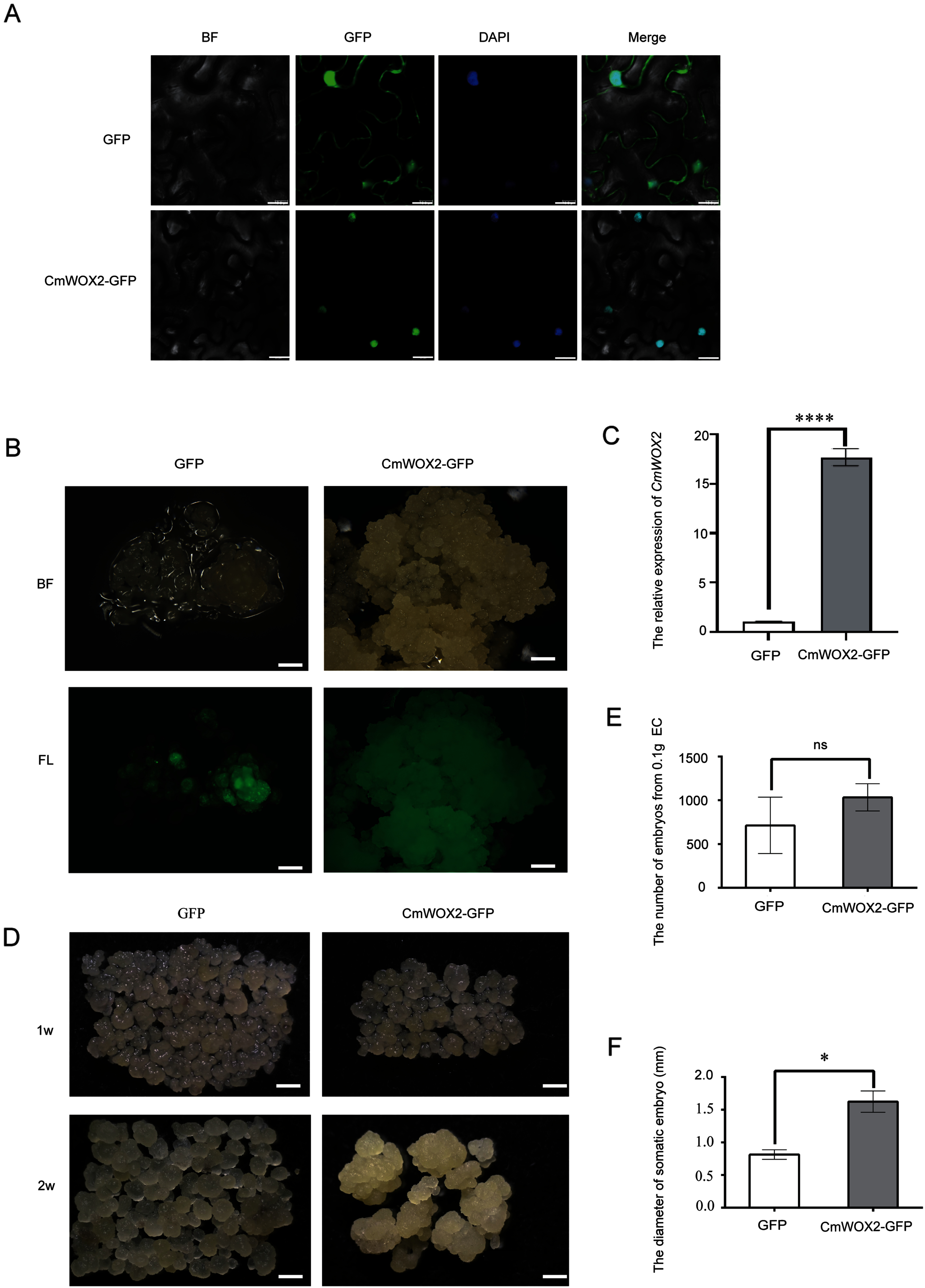
Figure 4. Subcellular localization of CmWOX2 and overexpression of *CmWOX2* in Chinese chestnut embryonic callus. A. Subcellular localization of CmWOX2. Bars: 10 µm. B. Visualization of *35S::CmWOX2-GFP* overexpressing and *35S::GFP* control callus using fluorescence Stereomicroscopy. Bars: 500 µm. C. Analysis of *CmWOX2* expression in overexpressing and control callus through qRT-PCR. D. Appearance of the *CmWOX2* overexpressing and control callus cultivated with E2 media for one and two weeks. Bars: 500 µm. E. Number of embryos formed in 0.1 g of *CmWOX2* overexpressing and control callus cultivated with E2 media for two weeks. F. Diameter of the embryogenic callus in *CmWOX2* overexpressing and control callus cultivated with E2 media for two weeks. Data represent mean±S.D. of 3 independent experiments. Student’s *t* test, **** *p*<0.0001, * *p*<0.05, ns indicate no statistically significant differences (*p*<0.05).

To explore the impact of *CmWOX2* on embryonic callus development, we generated an overexpression construct by fusing the *CmWOX2* coding sequence with GFP driven by 35S promoter (*35S::CmWOX2-eGFP*). Firstly, we confirmed the activity of this fused CmWOX2-eGFP protein by identifying its subcellular localization. We transiently expressed this construct in tobacco leaves alongside a control using *35S::eGFP* construct. Same as AtWOX2, the CmWOX2-eGFP fused protein was localized in the nucleus, while GFP alone was detected in both the nucleus and cell membrane (Figure 4A). Subsequently, we transformed *35S::CmWOX2-eGFP* and *35S::eGFP* into Chinese chestnut callus. The overexpressing (*35S::CmWOX2-eGFP*) callus lines and the control (*35S::eGFP*) lines were identified by visualizing GFP fluorescence (Figure 4B) and confirmed by assessing the relative expression of *CmWOX2* through qRT-PCR. The results demonstrated a 17 fold higher relative expression of *CmWOX2* in the *CmWOX2*-*GFP* lines compared to the *GFP* lines (Figure 4C).

The successful induction of *CmWOX2* expression in the embryonic callus enabled the application of *35S::CmWOX2-eGFP* for functional studies. To this end, we monitored the development of the embryos from overexpressing (*35S::CmWOX2-eGFP*) lines and the control (*35S::eGFP*) lines of similar size. After one week of cultivated in E2 (Embryo development liquid medium), the growth status appeared comparable between *CmWOX2* overexpressing and control callus (Figure 4D). However, after two weeks, despite the absence of significant differences (*p*<0.05) in the number of embryos formed within 0.1 g of callus in *GFP* lines (713.69±322.25) compared with the *CmWOX2*-e*GFP* lines (1033.35±154.94) (Figure 4E), the somatic embryo diameter of *CmWOX2*-e*GFP* lines (1.624±0.163 mm) was significantly (*p*<0.05) larger than that of *GFP* lines (0.812±0.072 mm) (Figure 4F). These findings suggested that the overexpression of *CmWOX2* enhances the embryogenic competence of callus by increasing embryo size.

### Exploring the role of *CmWOX2* in somatic embryogenesis across different varieties of *C. mollissima*

**Figure 5:**
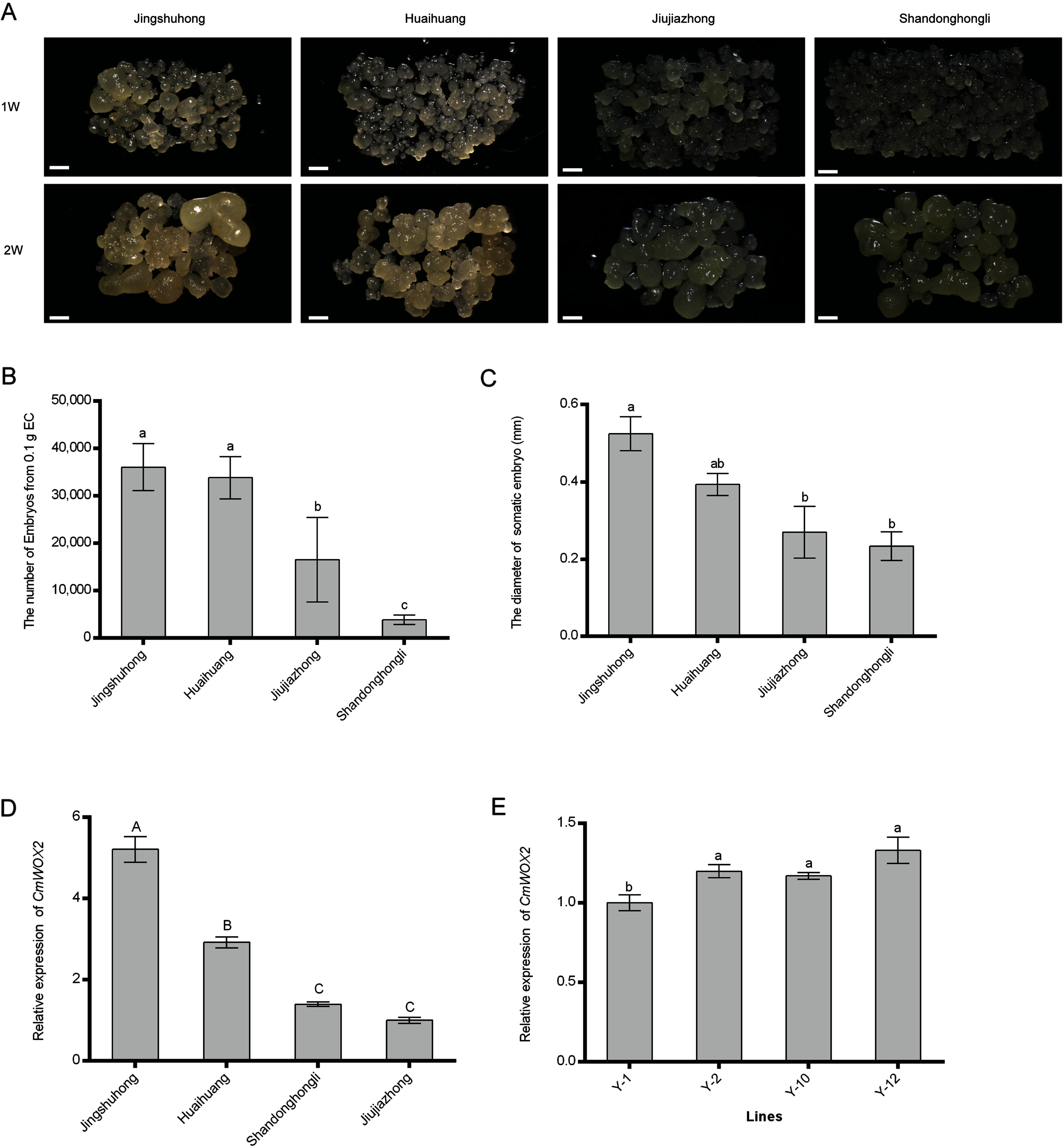
Figure 5. Somatic embryogenic capacity of different embryogenic cell lines. A. Somatic embryos of different embryogenic cell lines after E2 liquid suspension culture for 2 weeks. B. The number of somatic embryos. C. The average diameter of the somatic embryo. D. The expression of *CmWOX2* at EC stage in *C. mollissima* varieties lines. E. The expression of *CmWOX2* at EC stage in different lines. Bars: 500 µm. Data represent mean±S.D. of 3 independent experiments. Student’s *t* test, lowercase letters indicate statistically different groups, student’s *t* test, *p*<0.05, uppercase letters indicate statistically different groups, student’s *t* test, *p*<0.01.

To ascertain the function conservation of *CmWOX2* across different genetic backgrounds during somatic embryogenesis in *C. mollissima*, we conducted a comparative study involving various *C. mollissima* varieties and callus lines (Y- lines) exhibiting diverse embryonic competences, all derived from a single variety (Li et al. 2022). Initially, we assessed the development of somatic embryos from distinct *C. mollissima* varieties, include ‘Jingshuhong’, ‘Huaihuang’, ‘Jiujiazhong’ and ‘Shandonghongli’, following a two-week liquid suspension culture in E2 medium. Our findings revealed that ‘Jingshuhong’ and ‘Huaihuang’ produced a significantly (*p*<0.05) greater number of somatic embryos compare to ‘Jiujiazhong’ and ‘Shandonghongli’ (Figure 5A, B). Moreover, the average diameters of somatic embryos of ‘Jingshuhong’ were significantly (*p*<0.05) larger than those from ‘Jiujiazhong’ and ‘Shandonghongli’ (Figure 5A, C), indicating higher embryogenic competence in the former two varieties. Subsequent analysis of *CmWOX2* expression levels in these varieties and Y-callus lines during EC stage revealed that *CmWOX2* expression in ‘Jingshuhong’ and ‘Huaihuang’ was at least threefold higher than in ‘Jiujiazhong’ and ‘Shandonghongli’ (Figure 5D). Furthermore, *CmWOX2* exhibit significantly higher expression in in Y-2, Y-10 and Y-12 lines compared to Y-1 (Figure 5E). Correspondingly, the average diameters of somatic embryos from Y-2, Y-10 and Y-12 lines were significantly (*p*<0.01) larger than those from Y-1 (Li et al. 2022). These observations corroborate the pivotal role of CmWOX2 in facilitating somatic embryogenesis across a spectrum of genetic background in *C. mollissima*.

## Discussion

In this study, we have identified seven members of the Chinese chestnut *WUS* gene family. Among these seven genes, we pinpointed the *WOX2* homolog gene in Chinese chestnut (*CmWOX2*) and demonstrated its crucial role during the embryonic callus stage of SE. Overexpressing this gene enhanced the embryonic capability of chestnut callus, leading to a higher incidence of somatic embryo formation. Our result indicated *CmWOX2* is functionally conserved with *AtWOX2* in regulating SE process. Overexpression of *WUS* gene has been reported to increase the rate of SE or enhance somatic embryo development in various species, including *N. tabacum* (Rashid et al. 2007), *Coffea canephora* (Arroyo-Herrera et al. 2008), *Capsicum chinense* (Solís-Ramos et al. 2009), *Picea glauca* (Klimaszewska et al. 2010), *Gossypium hirsutum* (Bouchabké-Coussa et al. 2013), *Pinus pinaster* (Hassani et al. 2022). Therefore, *CmWOX2* emerges as a potential tool for expediting the SE process in chestnut.

In Arabidopsis, the *WUS* and *WOX5* genes are expressed in the organizing-center cells of the shoot and root apical meristem, respectively, plays a crucial role in maintaining stem-cell function (Burkart et al. 2022; Sarkar et al. 2007). Our phylogenetic analysis indicated that *CmWOX5* is the closest *AtWOX5* homolog gene and is allocated in the WUS clade. While *WOX7* regulates the number of lateral root primordia (Kong et al. 2020), a closely related homolog is absent in the Chinese chestnut genomes. This suggests that *CmWOX5* may be involved in both lateral root and root apical meristem development in Chinese chestnut. Furthermore, we identified two genes, *CmWUSa* and *CmWUSb*, as the closest *AtWUS* homolog. The expansion of *WUS* gene is not unique to chestnut and has also been reported in maize, specifically *ZmWUS1* and *ZmWUS2* (Nardmann and Werr 2006). These genes are involved in reproductive meristem and leaf primordia development, unlike their roles in Arabidopsis. Whether *CmWUSa*/*b* genes function similarly to *ZmWUS1*/*2* or participate in both reproductive and vegetative apical meristem development in chestnut requires further investigation. The *AtWOX1*/*6* clade, normally absent in monocot genomes, such as *Anana comosus*, *Musa acuminata*, *Oryza sativa*, *Phalaenopsis equestris*, *Phoenix dactylifera*, *Triticum aestivum* and *Zea mays* (Haecker et al. 2004; Wu et al. 2019), only revealed the presence of *CmWOX1* as the closest *AtWOX1* homolog and no *WOX6* gene was detected in the Chinese chestnut genomes.

The *CmWOX2* gene is crucial for SE in Chinese chestnuts as it influences the diameter of embryonic callus. In the *CmWOX2i* line, the average embryonic callus diameter was smaller compared to the EV line, although the differences did not reach statistical significance at *p*<0.05. Additionally, overexpressing *CmWOX2* significantly increased the embryonic callus diameter in Chinese chestnut. The minor phenotypic effect of *CmWOX2i* line is likely attributed to the low efficiency of the *CmWOX2* RNAi construct, which could only reduce half of the original *CmWOX2* expression. In contrast, the overexpression construct, *35S::CmWOX2-GFP*, induced 17-fold increase in *CmWOX2* expression.

*CmWOX2* may indirectly influence the somatic embryo formation rate. Reduced *CmWOX2* expression led to a significantly decrease in the number of somatic embryos, while overexpressing *CmWOX2* increased the average number of somatic embryos formed, although this increase was not statistically significant. These results suggested that *WOX2* has a discernible impact on somatic embryo development. However, given that *WOX2* is primarily expressed in embryogenic callus, we speculated that the effect of *WOX2* on somatic embryo formation rate is more of an aftereffect resulting from the impaired development of embryogenic callus.

In chestnut, *CmWOX2* emerges as the sole highly expressed *WUS* gene during SE, and we confirmed its role in embryonic callus development by its regulation of callus diameter. This findings aligns with numerous studies indicating a correlation between *WOX2* transcript levels and the embryogenic capacity of the tissues (Gambino et al. 2011; Palovaara et al. 2010). Specifically, *WOX2* has been demonstrated to exhibit high expression during SE in various species, including *Arabidopsis*, *Picea abies* and *Vitis vinifera* (Gambino et al. 2011; Palovaara et al. 2010; Wang et al. 2020). Notably, in Arabidopsis, *wox2* mutant exhibited a reduced somatic embryo formation rate (Wang et al. 2020), and the overexpression of *WOX2* fostered the formation of somatic embryos (Kadokura et al. 2018). In maize, *WOX2* was found to promote the regeneration of transgenic embryonic callus (Ma et al. 2018).

Beyond *WOX2*, a large number of transcription factors (TFs) had been reported to enhance the SE process in various species, including *ethylene-responsive factor 107*, *AINTEGUMENTA-LIKE 5*, *BABY BOOM*, *PLETHORA 2*, *CLAVATA 1*, *LEAFY COTYLEDON 1*, *LEAFY COTYLEDON 2*, *FUSCA 3*, *and AGAMOUS-LIKE 15 and AGAMOUS-LIKE 18* (Boutilier et al. 2002; El Ouakfaoui et al. 2010; Elhiti et al. 2010; Horstman et al. 2017; Ikeda et al. 2020; Jha and Kumar 2018; Li et al. 2019; Mantiri et al. 2008; Thakare et al. 2008; Tsuwamoto et al. 2010; Zheng et al. 2013). These genes, including *WOX2*, present promising avenues for improving the efficiency of SE in Chinese chestnut through genetic transformation. They stand as valuable candidates for future studies, offering significant potential to advance research and accelerate breeding programs in chestnut.

## Abbreviations

CE: cotyledon-type embryos
EC: embryogenetic callus
E1: embryo initiation medium
GE: globular embryo
GFP: green fluorescent protein
HE: heart embryo
SE: somatic embryogenesis
6-BA: 6-benzylaminopurine
TE: torpedo embryo
2,4-D: 2,4-dichlorophenoxyacetic acid

## Description of Supplementary Files

Supplementary Table S1. All primer sequences used in this study

Supplementary Figure S1. Super 1300 vector

Supplementary Figure S2. pK7GWIWG2(II) RR-277 vector

## References

[RAndrade2005] Andrade GM, Merkle SA (2005) Enhancement of American chestnut somatic seedling production. *Plant Cell Rep* 24: 326–334 15789206 10.1007/s00299-005-0941-0

[RArroyo2008] Arroyo-Herrera A, Ku Gonzalez A, Canche Moo R, Quiroz-Figueroa FR, Loyola-Vargas VM, Rodriguez-Zapata LC, Burgeff D′Hondt C, Suárez-Solís VM, Castaño E (2008) Expression of *WUSCHEL* in *Coffea canephora* causes ectopic morphogenesis and increases somatic embryogenesis. *Plant Cell Tissue Organ Cult (PCTOC)* 94: 171–180

[d67e1538] Bouchabké-Coussa O, Obellianne M, Linderme D, Montes E, Maia-Grondard A, Vilaine F, Pannetier C (2013) *Wuschel* overexpression promotes somatic embryogenesis and induces organogenesis in cotton (*Gossypium hirsutum* L.) tissues cultured *in vitro*. *Plant Cell Rep* 32: 675–686 23543366 10.1007/s00299-013-1402-9

[RBoutilier2002] Boutilier K, Offringa R, Sharma VK, Kieft H, Ouellet T, Zhang L, Hattori J, Liu C-M, van Lammeren AAM, Miki BLA, et al. (2002) Ectopic expression of BABY BOOM triggers a conversion from vegetative to embryonic growth. *Plant Cell* 14: 1737–1749 12172019 10.1105/tpc.001941 PMC151462

[RBurkart2022] Burkart RC, Strotmann VI, Kirschner GK, Akinci A, Czempik L, Dolata A, Maizel A, Weidtkamp-Peters S, Stahl Y (2022) PLETHORA-WOX5 interaction and subnuclear localization control Arabidopsis root stem cell maintenance. *EMBO Rep* 23: e54105 35373503 10.15252/embr.202154105 PMC9171415

[RCorredoira2003] Corredoira E, Ballester A, Vieitez AM (2003) Proliferation, maturation and germination of *Castanea sativa* Mill. Somatic embryos originated from leaf explants. *Ann Bot (Lond)* 92: 129–136 10.1093/aob/mcg107 PMC4243632 12763755

[RElOuakfaoui2010] El Ouakfaoui S, Schnell J, Abdeen A, Colville A, Labbé H, Han S, Baum B, Laberge S, Miki B (2010) Control of somatic embryogenesis and embryo development by AP2 transcription factors. *Plant Mol Biol* 74: 313–326 20798978 10.1007/s11103-010-9674-8 PMC2952763

[RElhiti2010] Elhiti M, Tahir M, Gulden RH, Khamiss K, Stasolla C (2010) Modulation of embryo-forming capacity in culture through the expression of Brassica genes involved in the regulation of the shoot apical meristem. *J Exp Bot* 61: 4069–4085 20729480 10.1093/jxb/erq222 PMC2935877

[RGambino2011] Gambino G, Minuto M, Boccacci P, Perrone I, Vallania R, Gribaudo I (2011) Characterization of expression dynamics of WOX homeodomain transcription factors during somatic embryogenesis in *Vitis vinifera.* *J Exp Bot* 62: 1089–1101 21127025 10.1093/jxb/erq349

[RGao2020] Gao Y, Sun J, Sun ZL, Xing Y, Zhang Q, Fang KF, Cao QQ, Qin L (2020) The MADS-box transcription factor *CmAGL11* modulates somatic embryogenesis in Chinese chestnut (*Castanea mollissima* Blume). *J Integr Agric* 19: 1033–1043

[RHaecker2004] Haecker A, Gross-Hardt R, Geiges B, Sarkar A, Breuninger H, Herrmann M, Laux T (2004) Expression dynamics of *WOX* genes mark cell fate decisions during early embryonic patterning in *Arabidopsis thaliana.* *Development* 131: 657–668 14711878 10.1242/dev.00963

[RHao2019] Hao Q, Zhang L, Yang Y, Shan Z, Zhou XA (2019) Genome-wide analysis of the WOX gene family and function exploration of *GmWOX18* in soybean. *Plants (Basel)* 8(7): 215–232 31373320 10.3390/plants8070215 PMC6681341

[RHassani2022] Hassani SB, Trontin JF, Raschke J, Zoglauer K, Rupps A (2022) Constitutive overexpression of a conifer *WOX2* homolog affects somatic embryo development in *Pinus pinaster* and promotes somatic embryogenesis and organogenesis in *Arabidopsis* seedlings. *Front Plant Sci* 13: 838421 35360299 10.3389/fpls.2022.838421 PMC8960953

[RHorstman2017] Horstman A, Bemer M, Boutilier K (2017) A transcriptional view on somatic embryogenesis. *Regeneration (Oxf)* 4: 201–216 29299323 10.1002/reg2.91 PMC5743784

[RIkeda2020] Ikeda M, Takahashi M, Fujiwara S, Mitsuda N, Ohme-Takagi M (2020) Improving the efficiency of adventitious shoot induction and somatic embryogenesis via modification of *WUSCHEL* and *LEAFY COTYLEDON 1.* *Plants (Basel)* 9: 1434 33113787 10.3390/plants9111434 PMC7692810

[RJha2018] Jha P, Kumar V (2018) *BABY BOOM (BBM)*: A candidate transcription factor gene in plant biotechnology. *Biotechnol Lett* 40: 1467–1475 30298388 10.1007/s10529-018-2613-5

[RKadokura2018] Kadokura S, Sugimoto K, Tarr P, Suzuki T, Matsunaga S (2018) Characterization of somatic embryogenesis initiated from the Arabidopsis shoot apex. *Dev Biol* 442: 13–27 29709600 10.1016/j.ydbio.2018.04.023

[RKlimaszewska2010] Klimaszewska K, Pelletier G, Overton C, Stewart D, Rutledge RG (2010) Hormonally regulated overexpression of Arabidopsis *WUS* and conifer *LEC1* (*CHAP3A*) in transgenic white spruce: Implications for somatic embryo development and somatic seedling growth. *Plant Cell Rep* 29: 723–734 20424847 10.1007/s00299-010-0859-z

[RKong2020] Kong J, Martin-Ortigosa S, Finer J, Orchard N, Gunadi A, Batts LA, Thakare D, Rush B, Schmitz O, Stuiver M, et al. (2020) Overexpression of the transcription factor *GROWTH-REGULATING FACTOR5* improves transformation of dicot and monocot species. *Front Plant Sci* 11: 572319 33154762 10.3389/fpls.2020.572319 PMC7585916

[RLi2019] Li K, Wang J, Liu C, Li C, Qiu J, Zhao C, Xia H, Ma C, Wang X, Li P (2019) Expression of *AtLEC2* and *AtIPTs* promotes embryogenic callus formation and shoot regeneration in tobacco. *BMC Plant Biol* 19: 314 31307397 10.1186/s12870-019-1907-7 PMC6633698

[RLi2022] Li XW, Sun ZL, Gao YR, Ge JY, Tian Y, Liu B, Sun S, Fang K, Qin L, Cao Q (2022) A strategy for establishing an efficient somatic embryo regeneration system in *Castanea mollissima* Blume. *Plant Cell Tissue Organ Cult (PCTOC)* 150: 299–312

[RLu2017] Lu D, Wei W, Zhou W, McGuigan LD, Ji FY, Li X, Xing Y, Zhang Q, Fang KF, Cao QQ, et al. (2017) Establishment of a somatic embryo regeneration system and expression analysis of somatic embryogenesis-related genes in Chinese chestnut (*Castanea mollissima* Blume). *Plant Cell Tissue Organ Cult (PCTOC)* 130: 601–616

[RMa2018] Ma L, Liu M, Yan Y, Qing C, Zhang X, Zhang Y, Long Y, Wang L, Pan L, Zou C, et al. (2018) Genetic dissection of maize embryonic callus regenerative capacity using Multi-Locus Genome-Wide Association studies. *Front Plant Sci* 9: 561 29755499 10.3389/fpls.2018.00561 PMC5933171

[RMantiri2008] Mantiri FR, Kurdyukov S, Lohar DP, Sharopova N, Saeed NA, Wang XD, Rose RJ (2008) The transcription factor *MtSERF1* of the ERF subfamily identified by transcriptional profiling is required for somatic embryogenesis induced by auxin plus cytokinin in *Medicago truncatula*. *Plant Physiol* 146: 1622–1636 18235037 10.1104/pp.107.110379 PMC2287338

[RMerkle1991] Merkle SA, Wiecko AT, Watson-Pauley BA (1991) Somatic embryogenesis in American chestnut. *Can J For Res* 21: 1698–1701

[RMiller2014] Miller AC, Woeste KE, Anagnostakis SL, Jacobs DF (2014) Exploration of a rare population of Chinese chestnut in North America: Stand dynamics, health and genetic relationships. *AoB Plants* 6: plu065 25336337 10.1093/aobpla/plu065 PMC4243075

[RNardmann2006] Nardmann J, Werr W (2006) The shoot stem cell niche in angiosperms: Expression patterns of *WUS* orthologues in rice and maize imply major modifications in the course of mono- and dicot evolution. *Mol Biol Evol* 23: 2492–2504 16987950 10.1093/molbev/msl125

[RPalovaara2010] Palovaara J, Hallberg H, Stasolla C, Hakman I (2010) Comparative expression pattern analysis of WUSCHEL-related homeobox 2 (*WOX2*) and *WOX8*/*9* in developing seeds and somatic embryos of the gymnosperm *Picea abies*. *New Phytol* 188: 122–135 20561212 10.1111/j.1469-8137.2010.03336.x

[RRashid2007] Rashid SZ, Yamaji N, Kyo M (2007) Shoot formation from root tip region: A developmental alteration by *WUS* in transgenic tobacco. *Plant Cell Rep* 26: 1449–1455 17426979 10.1007/s00299-007-0342-7

[RRaza2019] Raza G, Singh MB, Bhalla PL (2019) Somatic embryogenesis and plant regeneration from commercial soybean cultivars. *Plants (Basel)* 9: 38 31881730 10.3390/plants9010038 PMC7020241

[RSarkar2007] Sarkar AK, Luijten M, Miyashima S, Lenhard M, Hashimoto T, Nakajima K, Scheres B, Heidstra R, Laux T (2007) Conserved factors regulate signalling in *Arabidopsis thaliana* shoot and root stem cell organizers. *Nature* 446: 811–814 17429400 10.1038/nature05703

[d67e2151] Solís-Ramos LY, González-Estrada T, Nahuath-Dzib S, Zapata-Rodriguez LC, Castaño E (2009) Overexpression of WUSCHEL in *C. chinense* causes ectopic morphogenesis. *Plant Cell Tissue Organ Cult (PCTOC)* 96: 279–287

[RSparkes2006] Sparkes IA, Runions J, Kearns A, Hawes C (2006) Rapid, transient expression of fluorescent fusion proteins in tobacco plants and generation of stably transformed plants. *Nat Protoc* 1: 2019–2025 17487191 10.1038/nprot.2006.286

[RSun2020] Sun ZL, Li X, Zhou W, Yan J, Gao Y, Li XW, Sun JC, Fang KF, Zhang Q, Xing Y, et al. (2020) Agrobacterium-mediated genetic transformation of Chinese chestnut (*Castanea mollissima* Blume). *Plant Cell Tissue Organ Cult (PCTOC)* 140: 95–103

[RThakare2008] Thakare D, Tang W, Hill K, Perry SE (2008) The MADS-domain transcriptional regulator *AGAMOUS-LIKE15* promotes somatic embryo development in Arabidopsis and soybean. *Plant Physiol* 146: 1663–1672 18305206 10.1104/pp.108.115832 PMC2287341

[RTsuwamoto2010] Tsuwamoto R, Yokoi S, Takahata Y (2010) Arabidopsis EMBRYOMAKER encoding an AP2 domain transcription factor plays a key role in developmental change from vegetative to embryonic phase. *Plant Mol Biol* 73: 481–492 20405311 10.1007/s11103-010-9634-3

[RvanderGraaff2009] van der Graaff E, Laux T, Rensing SA (2009) The WUS homeobox-containing (WOX) protein family. *Genome Biol* 10: 248 20067590 10.1186/gb-2009-10-12-248 PMC2812940

[RVidal2010] Vidal N, Mallón R, Valladares S, Meijomín AM, Vieitez AM (2010) Regeneration of transgenic plants by *Agrobacterium*-mediated transformation of somatic embryos of juvenile and mature *Quercus robur*. *Plant Cell Rep* 29: 1411–1422 20972795 10.1007/s00299-010-0931-8

[RWang2020] Wang FX, Shang GD, Wu LY, Xu ZG, Zhao XY, Wang JW (2020) Chromatin accessibility dynamics and a hierarchical transcriptional regulatory network structure for plant somatic embryogenesis. *Dev Cell* 54: 742–757.e8 32755547 10.1016/j.devcel.2020.07.003

[RWang2021] Wang Y, Li HL, Zhou YK, Guo D, Zhu JH, Peng SQ (2021) Transcriptomes analysis reveals novel insight into the molecular mechanisms of somatic embryogenesis in *Hevea brasiliensis*. *BMC Genomics* 22: 183 33711923 10.1186/s12864-021-07501-9 PMC7953812

[RWu2019] Wu CC, Li FW, Kramer EM (2019) Large-scale phylogenomic analysis suggests three ancient superclades of the WUSCHEL-RELATED HOMEOBOX transcription factor family in plants. *PLoS One* 14: e0223521 31603924 10.1371/journal.pone.0223521 PMC6788696

[RXing2019] Xing Y, Liu Y, Zhang Q, Nie XH, Sun Y, Zhang ZY, Li HC, Fang KF, Wang GP, Huang HW, et al. (2019) Hybrid de novo genome assembly of Chinese chestnut (*Castanea mollissima*). *Gigascience* 8: giz112 31513707 10.1093/gigascience/giz112 PMC6741814

[RXing1999] Xing Z, Powell WA, Maynard CA (1999) Development and germination of American chestnut somatic embryos. *Plant Cell Tissue Organ Cult (PCTOC)* 57: 47–55

[RZheng2013] Zheng Q, Zheng Y, Perry SE (2013) Decreased *GmAGL15* expression and reduced ethylene synthesis may contribute to reduced somatic embryogenesis in a poorly embryogenic cultivar of *Glycine max*. *Plant Signal Behav* 8: e25422 23838957 10.4161/psb.25422 PMC4002625

