# Supplementary material for: CmWOX2 modulates somatic embryogenesis in Chinese chestnut (Castanea mollissima Blume)
Source: Plant Biotechnol (Tokyo). 2024 Dec 25;41(4):375–85. doi: 10.5511/plantbiotechnology.24.0527a (PMC11897729; doi:10.5511/plantbiotechnology.24.0527a)
Supplement: Supplementary Data [file plantbiotechnology-41-4-24.0527a-s001.pdf]

The following Supporting Information is available for this article:

Supplementary Table S1 All primer sequences used in this study

primer ID	Primer Sequence (5’ - 3’)	Purpose
CmWOX2-F	ATGGAGGGTGATAAT	Cloning CmWOX2 coding region
CmWOX2-R	CAAGGTTCCTGTGAAAGTGACTG	Cloning CmWOX2 coding region
1300-*CmWOX2*-F	CTCGATACACCAAATCGACTCTAGAATGGAGGGTGATAAT	Cloning CmWOX2 coding region into Super 1300 vector
1300-*CmWOX2*-R	CTCGCCCTTGCTCACCATGGTACCGGTTCCTGTGAAAGTGACTG	Cloning CmWOX2 coding region into Super 1300 vector
CmWOX2i-F	CCTCCTTGCCCAAATGTTCT	Cloning CmWOX2 coding region into pK7GWIWG2(II) RR-277 vector
CmWOX2i-R	TCCTTGTAAAATTCCTGTTG	Cloning CmWOX2 coding region into pK7GWIWG2(II) RR-277 vector
CmWOX2-qF	CTCCTCCTTGCCCAAATGT	qPCR of *CmWOX2*
CmWOX2-qR	GCACCTTGGAATGTTGGTATTG	qPCR of *CmWOX2*

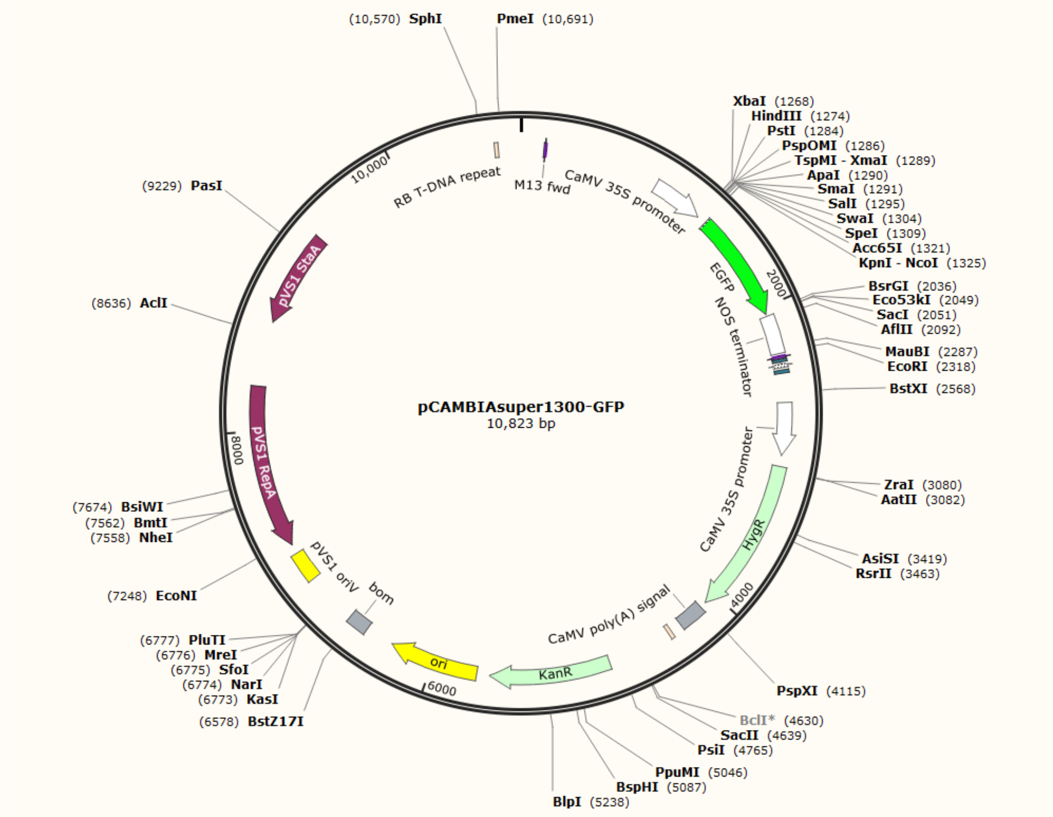

Supplementary Figure S1 Super 1300 vector

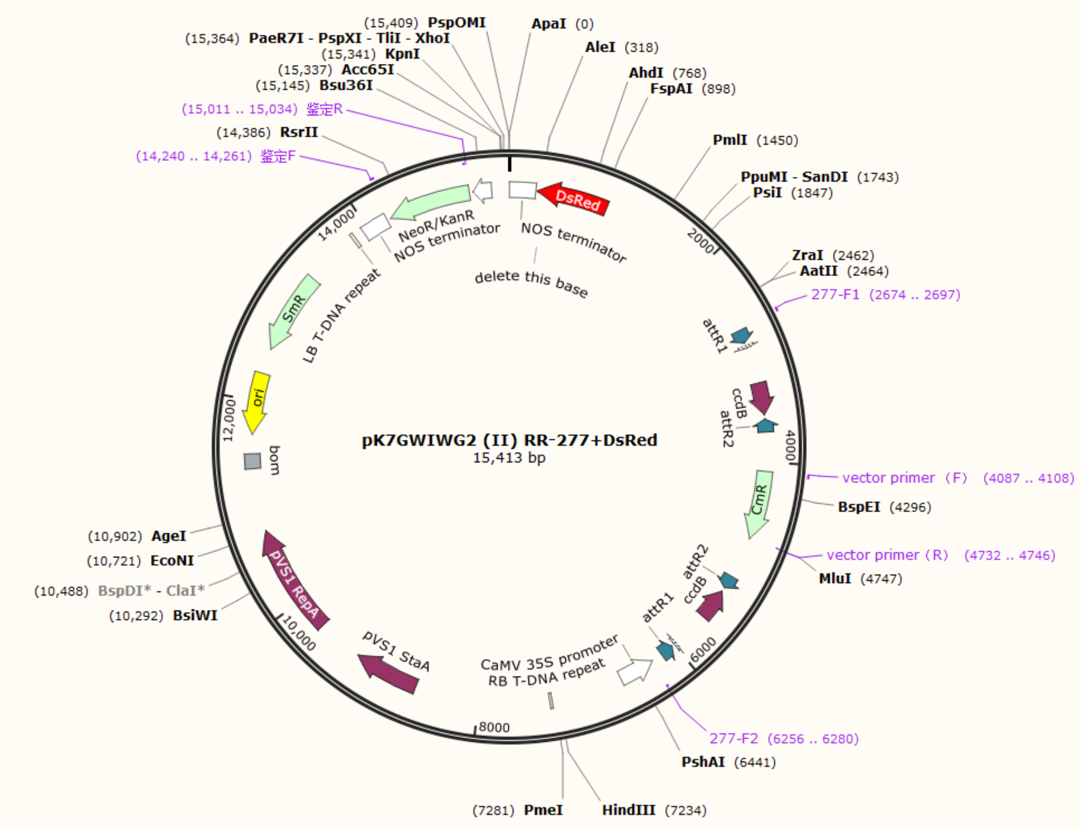

Supplementary Figure S2 pK7GWIWG2(II) RR-277 vector
